# Supporting Student Learning Needs in Tertiary Education: Institutional Support Structures Based on the Institutional Support Questionnaire

**DOI:** 10.3390/bs12080277

**Published:** 2022-08-11

**Authors:** Lyndon Lim, Yan Yin Ho

**Affiliations:** Teaching & Learning Centre, Singapore University of Social Sciences, Singapore 599494, Singapore

**Keywords:** higher education, tertiary education, questionnaire validation, learning support

## Abstract

This article presents and focuses on the Institutional Support Questionnaire (ISQ) that was developed and validated to complement the Learning Needs Questionnaire (LNQ). While the LNQ, validated and published earlier, assessed students’ perceived learning needs, the ISQ assesses students’ psychological perspectives of their institution, particularly how they perceive their institution supports their learning. Both questionnaires work in tandem to support resource optimisation efforts in establishing targeted academic support structures within teaching-focused tertiary institutions. This study found that the 42-item ISQ had adequate psychometric properties and that institutional support could be represented by four factors (i.e., academic competency support, teaching practices, tutors’ characteristics, and use of technology in instruction) that reflected in large part the factors characterised by the LNQ (i.e., perceived academic competency, time management, preferred tutors’ characteristics, and use of technology). Practical applications of the use of both the ISQ and LNQ (i.e., how both could be applied in a tertiary education setting to identify perceived students’ learning needs and whether an institution is providing adequate support to meet these needs) and limitations on their use are discussed.

## 1. Introduction

Supporting student learning is a mission naturally entrusted to all educational institutions. This is not an easy feat, considering that multiple moderators can impact a student’s academic success. In this regard, an assessment of learning needs is critical in that information derived from the assessment would inform how institutional support structures could be developed and optimised in order to motivate students toward academic success. This paper discusses the development and validation of a questionnaire, the Institutional Support Questionnaire (ISQ), that intends to, noting the finite nature of resources, support resource optimisation efforts in establishing targeted academic support structures within teaching-focused tertiary education institutions.

Notwithstanding other factors such as qualifications (e.g., undergraduate, postgraduate) and the nature (e.g., part-time, full-time) and delivery mode (e.g., full online, blended, face-to-face) of the programme, the amount of time a student spends at an institution is finite. By this token, educators should preferably, within all practical means, optimise students’ learning as much as possible within this time [1]. The impetus to achieve this drives institutions toward not only more fitting instructional practices but more optimal types of academic support afforded for its students. Study skill interventions are a typical type of academic support provided to students with the view that developing these study skills would have a positive impact on students’ academic performance; studies and meta-analyses by scholars have found these interventions generally effective [2,3]. Nonetheless, nuances in the efficacy of these interventions have been detected depending on whether: (1) the interventions were focusing on a single learning need, (2) the interventions adopted a multipronged approach where several learning needs were addressed, or (3) the interventions were metacognitive in nature. The effects of these interventions also differed when taking into account whether the interventions aimed to enhance performance that was closely related to (i.e., near transfer, which showed better results) or distantly related to (i.e., far transfer) the interventions. The current body of knowledge associated with study skill interventions is that for such interventions to be effective, the interventions should also incorporate metacognitive awareness and consider with caution the learning context.

Another study by King [4] identified two approaches to providing student support that aimed to enhance student learning: (1) an institutional-level approach emphasising learning experience and (2) an approach focused on student characteristics. This meant that institutions tried to support students by providing high-quality learning experiences, such as providing spaces and avenues for faculty–student interactions, and providing service learning and volunteering opportunities. On the other hand, learning support that focused on student characteristics attempted to develop and enhance educational practices that pertained to student attributes. King postulated that student support was comprehensive only when learning experiences and student characteristics were aligned and linked, student characteristics were acknowledged, and learning experiences were designed with these characteristics in mind. 

Evidently, the body of research on academic support provided by institutions (e.g., study skill interventions, enhancement of learning experiences, etc.) has suggested strong interest in, as well as recognition of the importance of, academic support structures for students’ academic success. While an institution that constantly reflects its adequacies in supporting student learning demonstrates commitment to providing a conducive learning environment to students, it must also be realistic in the allocation of its resources for such support structures. After all, many education institutions are publicly funded and must demonstrate responsibility and prudence in how resources are utilised [5]. Hence, other than the consideration of effective support structures for students, it is critical to consider key stakeholders’ views during this endeavour, one of which is to consider students’ psychological perspectives (i.e., hearing from students themselves with regard to the types of support they perceive they are receiving and need). Students’ perspectives would be informative for institutions in facilitating decision making about what types of academic support structures to implement or prioritise. The development of the ISQ serves this need, with a view to helping institutions optimise their resources by revealing targeted academic support structures that their students need. 

### 1.1. Dynamic Student Profiles, Dynamic Expectations and Learning Needs

The profile of students within institutions is ever changing. Contrary to the typical impression of a university campus filled with fresh secondary school graduates (i.e., conventional university students), there has been increasing diversity on campus, with more returning to schools to obtain or further their tertiary education qualifications [6,7,8]. Considering international advocacy toward continuing and further education [9] and the influx of adult learners, institutions should not assume that adult learners’ learning needs, and hence the expectations that the institution should have of adult learners, are similar to those of conventional university students [10]. Adult learners are busy individuals who have different and multiple priorities that impact how they expect learning to take place and how they learn. While some adult learners may be able to immerse themselves in some learning experiences, expecting no less in terms of the experience by conventional university students, other adult learners may face constraints and may not be able to spend as much time on their learning [11]. Studies have also shown that adult learners tend to learn through formal teaching and learning settings in the classroom, while conventional university students benefit from informal learning experiences from campus activities other than the formal teaching and learning settings [12]. The seemingly established profile of a conventional university student seems to be changing as well, and an example of such a change is that conventional students are spending more time working, in part to fulfil credits (e.g., work-study qualifications) [13]. Given this, it is premature now to claim that conventional university students have ample time for studying as compared with adult learners. With such complexities and the inevitable strain on resources brought about by an ever-shifting social–economic–political climate (e.g., due to the COVID-19 pandemic), it is all the more critical that feedback be sought from students on a regular basis on whether institutions have been keeping up with supporting their needs. It is with this framing that this study presents the ISQ, which was developed and validated as a tool to support resource optimisation efforts in establishing targeted academic support structures within teaching-focused tertiary education institutions.

### 1.2. Contextualising the Need for the Institutional Support Questionnaire (ISQ)

The ISQ is intended for teaching-focused tertiary education institutions, such as the case institution in this study, to have a validated tool to solicit feedback from students about their perceptions of the institutional support they are receiving for their learning. Based in Singapore, the case institution at the time of writing was uniquely the only publicly funded tertiary education institution that championed lifelong learning [14]. The Singapore Ministry of Education earlier designated the case institution to cater more to adult learners than conventional university students in efforts to diversify the local tertiary education landscape. From science and technology, to humanities and behavioural sciences, to business, to human development, to law, the case institution offered a variety of undergraduate, postgraduate, and continuing education programmes that catered to working adults who want to pursue tertiary-level education. Naturally, the case institution had about 75% of its student population being adult learners enrolled in programmes that were usually part-time in nature, with classes arranged in the evenings or weekends to cater to adult learners’ schedules and other commitments. To afford greater flexibility, the case institution also offered courses in a variety of face-to-face, blended, or fully online modes.

Based on Messick’s first facet of validity evidence [15], content appropriateness, it is critical to consider the context in which a test or questionnaire is to be deployed. By token of the context described, the absence of a questionnaire in the Singapore tertiary education institution context, and the case institution having a unique student population (i.e., 75% adult learners), the ISQ had to be developed for its intended purpose.

## 2. Methodology

The ISQ was developed based on the measurement of latent variables and classical measurement theory as described by DeVellis [16]. Essentially, each subscale is measured unidimensionally by a number of items, upon which a score is computed and claims are made. Three stages, as recommended by Messick [15], were undertaken for this study. Stage 1 involved developing items, with a focus on content appropriateness. Stage 2 involved administering the ISQ to collect data, and Stage 3 involved analyses to establish the factorial structure of the ISQ.

*Stage 1*

A separate questionnaire, the Learning Needs Questionnaire (LNQ) by Ho and Lim [17], was developed and validated earlier for the case institution to assess what students perceived of themselves. Items in the ISQ were developed with reference to what students perceived of the institution and in part to the results of the validation of the LNQ. This would allow the ISQ and LNQ to be complementary, such that information gathered from both questionnaires would be useful in supporting institutional efforts toward supporting students. Based on the literature review, there appeared to be a dearth of existing and complementary instrument(s) that had been developed via a positivist approach with adequate psychometric properties for claims suitable for the purpose of establishing student learning support structures at teaching-focused tertiary education institutions. In this regard, the ISQ items were primarily developed via a deductive research approach by reviewing existing nonvalidated measures of institutional support and the various intended dimensions within these measures. Along with this, inductive approaches were used when items were developed by drawing from academic and professional staff experience within a unit in the Office of the Provost responsible for planning and implementing academic support structures at the institutional level.

The initial item development stage saw the generation of 56 items representing how students perceived institutional support structures related to key domains such as academic competency support, learning resources, tutors’ teaching practices, and tutors’ characteristics. These items were developed in part with a view that tutors, which in the case institution meant the course instructors, were faces of the institution, as they interacted the most with students. Five of the fifty-six items were removed, as they were deemed inappropriate or confusing for respondents by an expert panel comprising a member of the institution senior management, a senior lecturer, a lecturer, and five professional staff under the Office of the Provost. Table 1 presents the reasons why these items were removed.

### 2.1. Rating Scale and Scoring

For the purposes of respondents’ familiarity and minimising potential confounds due to confusion, the ISQ employed a rating scale similar to that of the LNQ. Item responses were coded one to seven, with seven and one corresponding to strongly agree and strongly disagree, respectively.

*Stage 2*

Stage 2 was focused on data collection over a two-month period. Apart from email invitations seeking student respondents sent to all official student email addresses, digital signage boards across all lift lobbies in the case institution advertised the call for student respondents. Interested respondents would then access the ISQ via a hyperlink. To further encourage students to participate, the email invitation and digital advertisements specified that respondents who completed the questionnaire would be eligible to receive a randomly drawn prize. Ethical review and approval were waived for this study on the basis that this research was conducted in established or commonly accepted educational settings and involved normal educational practices. Informed consent was assumed when students voluntarily accepted the invite and responded by completing the ISQ.

### 2.2. Participants and Procedure

A total of 1178 participant responses were used to validate the ISQ (see Table 2). Responses with at least one missing value and duplicates were excluded.

*Stage 3*

Exploratory and confirmatory factor analyses (EFA and CFA) were used in stage 3. As described by DeVellis [16], EFA was used to establish the factorial structure of the ISQ as well as for item reduction based on statistical results rather than theory. CFA was then used to determine the extent to which the a priori factorial structure established through EFA represented the actual data [18]. For the purpose of cross-validation, the sample was partitioned, and one half was used for each of EFA and CFA. Each half of the sample was comparable in terms of gender, χ^2^ (1) = 0.01, *p* > 0.05; degree programme (part-/full-time undergraduate, postgraduate), χ^2^ (2) = 0.11, *p* > 0.05; years of study with the university, χ^2^ (5) = 1.43, *p* > 0.05; school respondent was from, χ^2^ (5) = 0.07, *p* > 0.05; age group, χ^2^ (5) = 0.26, *p* > 0.05; cumulative grade point average band, χ^2^ (7) = 3.03, *p* > 0.05; and highest educational qualification attained before matriculation, χ^2^ (4) = 3.49, *p* > 0.05. 

### 2.3. Exploratory Factor Analysis

Tests of normality (Mardia, Shapiro–Wilk, Kolmogorov–Smirnov, Cramer–von Mises, and Anderson–Darling) were undertaken to assess the suitability of EFA for the dataset. The dataset was considered suitable for EFA based on recommendations by Fuller and Hemmerle [19], given that the distributions of all items were modestly nonnormal; the skewness and kurtosis of each were also within the thresholds recommended for structural equation modelling [20]. Further to tests of normality, the Kaiser–Meyer–Olkin measure of sampling adequacy, was 0.95 and Barlett’s test of sphericity was significant (x^2^ (1081) = 33,452.69, *p* < 0.001). These metrics also supported the suitability of EFA for the dataset.

Cattell’s scree test, Kaiser’s eigenvalue criterion, Horn’s parallel analysis, and the amount of variance explained were considered in establishing the factorial structure. Four factors were identified, along with a reduction of eight items, as these had item loadings of less than 0.32 or were cross-loaded [18,21]. These factors and their corresponding variances explained were interpreted as tutors’ characteristics (TC, 79.26%), academic competency support (ACS, 9.82%), use of technology in instruction (TII, 7.97%), and teaching practices (TP, 2.95%). The interfactor correlations, means, and standard deviations and final factor loadings (after item reduction) are presented in Table 3 and Table 4, respectively.

### 2.4. Reliability

Reliability indices suggested sufficient internal consistency based on the thresholds recommended by Everitt [22], Kline [23], and Tucker and Lewis [24]. Cronbach’s α was 0.97, Tucker and Lewis’s reliability coefficient was 0.78, and item total correlations ranged from 0.43 to 0.81. In view of these, all items that were used for EFA were retained without further deletion. 

### 2.5. Confirmatory Factor Analysis

Based on the factors specified through the EFA, a four-factor measurement model of the ISQ was proposed. This model was then subjected to a hierarchical CFA to assess model fit and confirm the factorial structure of the instrument. As with the partitioned sample for EFA, the CFA sample was slightly nonnormal, and hence, the maximum likelihood with Satorra–Bentler (MLSB) scaled chi-square statistics for model fit that adjusted the chi-square statistic and standard errors for data nonnormality was used for more precise goodness-of-fit statistics [25,26,27].

Initial iterative runs of the CFA found few items that contributed to model misfit. Table 5 presents these items and suggestions why they might have contributed to model misfit.

With the removal of these five items, the model fit of the first-order four-factor model specified of the ISQ by the EFA was assessed to be acceptable based on recommended thresholds, indicators, and goodness-of-fit statistics per Brown [28], Hair et al. [18], and Hu and Bentler [29] (i.e., CFI ≥ 0.90; RMSEA < 0.08; SRMR < 0.08). Theoretically plausible models (i.e., one-factor model, second-order four-factor model) were also considered to ascertain the feasibility of the first-order four-factor model. Table 6 present results of the CFA. Based on the chi-square difference test, Schwartz Bayesian Criterion and Akaike Information Criterion, it could be suggested that the first-order four-factor model specified by the EFA but with five items removed was most appropriate for the ISQ.

All standardised loading estimates of the first-order four-factor model were significant (*p* < 0.001) and greater than 0.5; these suggested a factorial structure with practical significance [18]. With the exception of items ACS5 (loading 0.6), TP9 (loading 0.5), TP5 (loading 0.6), TC20 (loading 0.6), and TII6 (loading 0.4), all ISQ items had standardised loadings greater than 0.7; these further indicated a well-defined factorial structure.

The construct reliability (CR) of each factor was well above 0.7; this indicated that there was internal consistency and that the items consistently measured the same constructs. The average variance extracted (AVE) for each factor was also above 0.5, suggesting acceptable error variance and that no items should be further eliminated.

To investigate whether each factor was distinct from the others, the square root of the AVE was compared with the interfactor correlations as recommended by Fornell and Larcker [30] and Hair et al. [18]. It was established that all the factors were distinct from others, given that the square roots of the AVE estimates were greater than the interfactor correlations (see Table 7 and Table 8).

## 3. Discussion

The EFA and CFA suggested a first-order four-factor model to represent the ISQ, with a reduction of 14 items across stages 1 to 3 of this study. The four factors (i.e., tutors’ characteristics, academic competency support, use of technology in instruction, and teaching practices) would invariably inform how students perceived institutional support vis-à-vis their learning. Other than the *teaching practices*, the factors found for the ISQ in this study complemented three of the four factors identified by the LNQ (i.e., student preference of tutors’ characteristics, use of technology, perceived academic competency). Since the development of the ISQ and LNQ had been intended to be complementary in their efforts to help institutions establish the learning support required by their students, the following sections discuss how the factors identified in the ISQ and LNQ complemented each other (see Table 9 for factors).

### 3.1. Tutors’ Characteristics

While the LNQ measures student preferences of tutor characteristics, the ISQ measures what students go through (i.e., what their tutors’ characteristics were). Administered together (e.g., the LNQ followed by the ISQ), gaps between scores of the LNQ and ISQ in this area could indicate a disconnect in that what students preferred of tutors was inconsistent with the characteristics displayed by tutors. Students’ perception of the quality of instruction has been associated with tutor-related factors [31]. For example, students’ enjoyment of a class seemed to be mediated by students’ perception of an instructor’s enthusiasm [32]. More importantly, such a perception seemed to have an impact on student learning. A three-year study by Alshariff and Qi [33] showed that students’ motivation to learn was related to their perception of tutor-related factors. Despite these studies, though, there seem to have been limited studies on students’ perception of an ideal tutor [34]. Hence, it would be judicious for institutions to survey their students to understand their preferences for tutor-related characteristics. When the responses were compared with the responses regarding students’ actual experience of their tutors, institutions would then be better placed to assess whether students’ expectations have been met. In the event that there were a disconnect arising from responses to the ISQ, institutions would have relevant information to convey to tutors; this could be used in part to develop more meaningful tutor–student interactions that support learning. 

### 3.2. Academic Competency Support

*Academic competency support* (ACS) within the ISQ provides information on students’ perception of the support they receive from their institution in developing their academic skills (e.g., verbal presentation skills, critical thinking, etc.). Taken together with the perceived academic competency measure within the LNQ, which measures students’ perception of their own academic competency, information regarding the sufficiency of ACS could be obtained. As an example, if students reported a low perceived academic competency in their academic writing skills and concurrently reported a low perception of institutional support in the area of academic writing, this would be an indication that academic writing support was not sufficient within the institution and that resources should be directed to develop support in this area. 

The assessment of ACS provided by an institution has important implications for student retention. Whether in Tinto’s student integration model, which suggests academic and social experiences in college as determinants for academic persistence; Bean and Metzner’s student attrition model, which explains academic persistence of nontraditional students; or Rovai’s model of student persistence, which integrates both Tinto’s and Bean and Metzner’s models, the roles of academic experience and support provided by institutions still feature prominently as factors related to student retention [35]. Indeed, institutions have a moral obligation to provide the necessary support to afford their students the highest chance for success. This is especially important when public funds are used to finance operations [36]. In view of this, institutions should regularly seek feedback from their students about their experience of ACS, and the ACS factor of ISQ holds promise for this purpose. 

### 3.3. Use of Technology in Instruction

Given the advent of information technology in education, it is critical to learn how students view their institutions in terms of the use of technology in instruction, which is what the *use of technology in instruction* factor of the ISQ was intended to measure. This measure could also be compared with the LNQ’s *student use of technology* measure; a difference in scores could indicate that a student was IT savvy but receiving instruction that did not optimise the use of information technology for learning, whereas a high score achieved in both scales could indicate that the institution had prepared both students and tutors to function satisfactorily in a technology-assisted learning space.

Increased dependence on and use of technology in education have been keenly felt during the COVID-19 pandemic. With schools and tertiary education institutions locked down to prevent the spread of the virus, technology was heavily utilised to ensure that teaching and learning could continue [37]. With this, students and instructors were thrown into a new reality overnight, which included the need to be able to learn and teach effectively using relevant technologies. While the world is still grappling with containing the virus, students and instructors are now more familiar in the online teaching and learning space than they were during the beginning phases of the pandemic. This presents an opportune time to assess whether the quick pivot to online education has accelerated not only students’ information technology skills but instructors’ expertise in using technology to engage students, and the ISQ *use of technology in instruction* factor is timely for this purpose.

### 3.4. Teaching Practices

Based on the findings of this study, there was no direct association between the *teaching practices* factor and those of the LNQ. Nonetheless, teaching practices are the face of an education institution. Whether students receive quality teaching can be impacted by what goes on in the classrooms. In view of this, how students view institutional support in terms of teaching practices would provide institutions, including tutors, with information on potential areas for improvement. As an example, if the item “My tutor uses learning outcomes to guide students on what to learn” consistently returned a low rating of agreement, it would pay to investigate how tutors could level up in constructive alignment [38] so that teaching practices were consistent with intended learning outcomes. 

## 4. Limitations and Directions for Future Research

The ISQ was developed and validated to complement the LNQ for the case institution. As the validation was based on classical test theory and hence sample dependent, it would be most appropriate to examine the generalisability of the ISQ in tertiary education institutions that place a high value on teaching and have diverse student populations, as with the case institution. To further minimise sample dependency and provide a basis for exploring the additivity of scores, a Rasch analysis could be undertaken subsequently [26]. Nonetheless, the ISQ (along with the LNQ) presents a reference for tertiary education institutions to develop a comprehensive questionnaire that evaluates both students’ learning needs and their perceptions of how the institution may be meeting their needs. 

It is worth noting that the ISQ items were conceptualised before COVID-19 affected the experience of education. Therefore, there is a possibility that some items may not be sensitive to changes due to the pandemic. This calls for a review of the items before subsequent administration, as is normal practice when deploying questionnaires. 

## 5. Conclusions and Practical Implications

The development and validation of an instrument can be time and resource intensive. However, the processes are necessary so that the responses collected present valid and reliable measures. Based on the review of literature and the absence of a similar questionnaire in the Singapore context, the ISQ was developed and validated in the context of the educational experiences of students at the case institution. 

The advent of lifelong learning, with the aim to live well and cope with today’s ever-changing world, means that people are expected to return to school from time to time throughout life in order to upskill, reskill, or just learn for pleasure. Hence, education institutions should expect greater diversity within their student population, not only in terms of student characteristics (i.e., a mix of conventional fresh graduates and adult learners) but in terms of motivation for learning. Such diversity is undeniably also fuelled by the development of continuing education units and the potential of stackable credentials in institutions [39]. This calls for institutions to regularly survey their students to learn about how students perceive institutional support vis-à-vis their learning needs. In view of this, the ISQ, in parallel with the LNQ, holds promise as an instrument that can be readily deployed on a practical and large-scale basis in institutions for this purpose; the ISQ assesses students’ perceptions of institutional support of their learning, while the LNQ assesses their perceived learning needs. With the information gathered from these measures, institutions would be better placed to fulfil their responsibility and moral duty of providing students under their charge the opportunity for academic success. In a practical setting, this means that there must be targeted support structures to help both low- and higher-progress students to realise and maximise their potential. 

## Figures and Tables

**Table 1:** Items removed from original item pool.

Item Stem	Reason(s) for Removal
My tutor is able to incorporate students’ experiences to deepen learning.	It may be unclear how learning might be deepened by incorporating students’ experiences.
My tutor is able to engage students in the online learning environment.	Item precludes other learning environments that are also prevalent within the institution (e.g., face to face, blended).
My tutor provides feedback on assessments and assignments that help students to improve.	It is not clear whether it is the feedback or the assessment that is meant to help students to improve.
My tutor is empathetic to student learning needs.	Empathetic might not be understood by students enrolled in non-English disciplines. Item may also be perceived as similar to another item, “my tutor is helpful to students in addressing their learning needs”.
My tutor cares about student learning.	Item was unanimously considered vague.

**Table 2:** Profile of respondents.

Characteristics	Sample Partitioned for EFA		Sample Partitioned for CFA	
	*n*	%	*n*	%
Gender				
Male	253	43.0	255	43.3
Female	336	57.1	334	56.7
Degree programme				
Full-time undergraduate	150	25.5	147	25.0
Part-time undergraduate	416	70.6	417	70.8
Postgraduate	23	3.9	25	4.2
Years of study with the university				
Less than 1	157	26.7	149	25.3
1 to 2	171	29.0	184	31.2
3 to 4	218	37.0	220	37.4
5 to 6	37	6.3	30	5.1
7 to 8	5	0.9	5	0.9
More than 8	1	0.2	1	0.2
School				
A	9	1.5	10	1.7
B	133	22.6	132	22.4
C	244	41.4	244	41.4
D	102	17.3	101	17.2
E	8	1.4	8	1.4
F	93	15.8	94	16.0
Age group				
Below 25	244	41.4	243	41.3
25 to 30	191	32.4	196	33.3
31 to 40	86	14.6	84	14.3
41 to 50	46	7.8	44	7.5
51 to 60	16	2.7	17	2.9
Above 61	6	1.0	5	0.9
Cumulative grade point average band				
4.51 to 5.00	14	2.4	10	1.7
4.01 to 4.50	50	8.5	55	9.3
3.51 to 4.00	116	19.7	110	18.7
3.01 to 3.50	137	23.3	146	24.8
2.51 to 3.00	83	14.1	76	12.9
2.00 to 2.50	50	8.5	53	9.0
0 to 1.99	8	1.4	13	2.2
Not sure	131	22.2	126	21.4
Highest educational qualification before matriculation				
Postgraduate	17	2.9	11	1.9
Degree	48	8.2	55	9.3
Diploma	426	72.3	441	74.9
GCE A Level	74	12.6	63	10.7
GCE O Level	24	4.1	19	3.2

**Table 3:** Interfactor correlations, means, standard deviations, and Cronbach’s α.

ISQ	F1	F2	F3	*M*	*SD*	α
TC (F1)				5.54	0.92	0.81
ACS (F2)	0.42			4.87	1.00	0.85
TII (F3)	0.62	0.43		5.23	1.04	0.83
TP (F4)	0.62	0.54	0.56	5.11	0.94	0.81

**Table 4:** Final factor loadings.

Items	F1	F2	F3	F4
TC1. Is approachable	**0.92**	0.04	−0.03	−0.03
TC2. Is patient with students	**0.91**	0.03	0.00	−0.04
TC3. Is helpful to students in addressing their learning needs	**0.91**	0.04	0.01	−0.03
TC4. Respects students	**0.90**	0.08	−0.04	−0.12
TC5. Is dedicated to teaching	**0.90**	0.02	−0.04	0.05
TC6. Is enthusiastic in teaching	**0.87**	−0.04	0.03	0.08
TC7. Prepares their lessons well	**0.87**	−0.01	0.04	0.01
TC8. Explains clearly in lessons	**0.87**	−0.03	0.03	0.06
TC9. Motivates students to learn	**0.84**	0.06	0.04	0.04
TC10. Has strong subject knowledge	**0.83**	−0.16	0.04	0.09
TC11. Provides high-quality feedback	**0.82**	0.14	0.01	−0.03
TC12. Provides prompt feedback	**0.80**	0.14	0.04	−0.04
TC13. Provides sufficient learning materials	**0.80**	0.02	0.10	0.02
TC14. Delivers interesting lessons	**0.73**	0.03	0.08	0.09
TC15. Believes in students’ learning ability	**0.67**	0.04	0.13	0.09
TC16. Stimulates student thinking	**0.66**	−0.03	0.10	0.23
TC17. Is humorous in teaching	**0.66**	−0.02	0.08	0.12
TC18. Relates work or life experiences to concepts taught	**0.54**	−0.11	0.14	0.26
TC19. Sets high learning expectations	**0.43**	0.04	0.21	0.15
TC20. The learning materials (presentation slides, readings, activity sheets, etc.) prepared by my tutor help my learning	**0.41**	0.05	0.10	0.23
ACS1. My verbal presentation skills are developed by the resources, courses, and workshops provided by the university	0.00	**0.93**	−0.01	−0.12
ACS2. My critical thinking skills have been honed through the resources, courses, and workshops provided by the university	0.05	**0.83**	−0.09	0.04
ACS3. My academic writing needs are developed and supported by the resources, courses, and workshops provided by the university	0.11	**0.83**	0.02	−0.20
ACS4. I am able to develop my verbal presentation skills through tutors’ feedback and advice	−0.03	**0.75**	0.04	0.04
ACS5. My academic writing needs are developed and supported by tutors’ feedback and advice	0.14	**0.62**	−0.01	0.05
ACS6. My critical thinking skills have been honed through feedback from tutors	0.08	**0.60**	−0.13	0.31
ACS7. The university provided opportunities to hone my information technology skills	−0.05	**0.57**	0.23	0.09
ACS8. The university provided opportunities to hone my research	0.04	**0.56**	0.00	0.22
ACS9. My tutors helped me to be better at researching information for my academic work	−0.01	**0.54**	0.08	0.29
ACS10. My tutors taught me to be better at using information technology	−0.15	**0.52**	0.33	0.17
ACS11. The university provided opportunities to develop my self-directed learning skills	0.00	**0.44**	0.11	0.27
TII1. Uses technology to make learning more flexible	0.10	−0.04	**0.86**	0.09
TII2. Uses technology to positively enhance my learning experience	0.20	0.03	**0.85**	−0.03
TII3. Uses technology to enhance learning	0.16	0.01	**0.81**	−0.02
TII4. Is comfortable using technology to help my learning	0.20	−0.03	**0.79**	0.03
TII5. Makes technology an integral part of my learning experience	0.18	0.03	**0.78**	0.02
TII6. The prerecorded lectures are useful to my learning	0.04	0.21	**0.40**	−0.04
TII7. The classroom replay videos are useful to my learning	0.06	0.19	**0.35**	−0.03
TP1. My tutor is able to challenge students to broaden their perspectives	0.23	0.00	−0.09	**0.76**
TP2. My tutor is able to provide strategies to students to help them understand their learning	0.19	0.08	−0.07	**0.75**
TP3. My tutor is able to show students how theories are applied	0.28	−0.04	−0.09	**0.74**
TP4. My tutor encourages students to share their learning with peers	0.07	0.10	0.03	**0.60**
TP5. My tutor uses learning outcomes to guide students on what to learn	0.07	0.07	0.06	**0.59**
TP6. My tutor is able to use assessment to identify gaps in students’ learning	−0.04	0.23	0.10	**0.58**
TP7. My tutor uses assessment rubrics to guide students’ learning	−0.03	0.18	0.13	**0.50**
TP8. My tutor summarises main issues covered in each session at the end of each class	0.23	0.11	0.10	**0.39**
TP9. My tutor uses past year exam questions to guide students’ learning	0.08	0.08	0.09	**0.35**

**Table 5:** Items that contributed to model misfit.

Item	Possible Reasons for Misfit
TC4. Respects students	There could have been multiple interpretations of “respect” and how this might be an indicator of tutors’ characteristics.
ACS4. I am able to develop my verbal presentation skills through tutors’ feedback and advice	Presentations are normally marked as part of assessment, and hence, students might view the process as more evaluative than developmental. Further, the final assessment task in general is either a written exam or essay assignment.
ACS10. My tutors taught me to be better at using information technology	Learning support in terms of information technology is provided by a unit within the university rather than tutors.
TII7. The classroom replay videos are useful to my learning	The replay videos could have helped some but not other groups, particularly if the videos did not capture discussions in discussion-heavy seminars.
TP7. My tutor uses assessment rubrics to guide students’ learning	It is not common practice that tutors inform students that rubrics are used for marking.

**Table 6:** CFA goodness-of-fit indicators.

Model	χ^2^	χ^2^*_diff_*	*df*	χ^2^*/df*	CFI	RMSEA	SRMR	AIC	SBC
One-factor	4379.69 *		819	5.35	0.70	0.09	0.09	4547.69	4915.48
Second-order four-factor	1925.48 *	2454.21 *	815	2.36	0.91	0.05	0.05	2101.48	2486.78
First-order four-factor	1902.81 *	2476.88 * 22.67 *^,#^	813	2.34	0.91	0.05	0.05	2082.81	2476.86

*Note:*. χ^2^ = Satorra–Bentler scaled chi-square statistic; χ^2^*_diff_* was computed relative to the first-order one-factor model; *df* = degrees of freedom; CFI = comparative fit index; RMSEA = root mean square error of approximation; SRMR = standardised root mean square; AIC = Akaike information criterion; SBC= Schwarz Bayesian criterion. ^#^ refers to x^2^*_diff_* relative to the second-order four-factor model. * *p* < 0.001.

**Table 7:** Standardised loadings, AVE, and CR coefficients of the first-order four-factor model.

Construct and Items	Standardised Loading	AVE	CR
TC (F1)		0.64	0.89
TC1	0.81		
TC2	0.78		
TC3	0.84		
TC5	0.85		
TC6	0.86		
TC7	0.83		
TC8	0.84		
TC9	0.87		
TC10	0.76		
TC11	0.83		
TC12	0.81		
TC13	0.82		
TC14	0.85		
TC15	0.83		
TC16	0.85		
TC17	0.73		
TC18	0.72		
TC19	0.67		
TC20	0.65		
ACS (F2)		0.53	0.93
ACS1	0.75		
ACS2	0.79		
ACS3	0.76		
ACS5	0.61		
ACS6	0.73		
ACS7	0.74		
ACS8	0.77		
ACS9	0.73		
ACS11	0.68		
TII (F3)		0.73	0.96
TII1	0.94		
TII2	0.92		
TII3	0.89		
TII4	0.93		
TII5	0.92		
TII6	0.39		
TP (F4)		0.51	0.96
TP1	0.81		
TP2	0.83		
TP3	0.80		
TP4	0.69		
TP5	0.65		
TP6	0.67		
TP8	0.69		
TP9	0.54		

**Table 8:** Distinctiveness of factors.

Construct	TC	ACS	TII	TP
TC	0.89 *			
ACS	0.58	0.85 *		
TII	0.72	0.54	0.91 *	
TP	0.79	0.70	0.64	0.84 *

* refers to the square root of AVE.

**Table 9:** Factors from the ISQ and LNQ.

ISQ Factors	LNQ Factors
Tutors’ Characteristics *	Preferred Tutors’ Characteristics *
Academic Competency Support ^#^	Perceived Academic Competency ^#^
Use of Technology in Instruction ^@^	Use of Technology ^@^
Teaching Practices	Time Management

*^,#,@^ Factors that could be used complementarily with each other.

## Data Availability

Data available on request because of privacy and ethical restrictions.
